# Supplementary material for: The emergence of magnetic ordering at complex oxide interfaces tuned by defects
Source: Nat Commun. 2020 Jul 20;11:3650. doi: 10.1038/s41467-020-17377-0 (PMC7371687; doi:10.1038/s41467-020-17377-0)
Supplement: Supplementary file 1 — Supplementary Information [file 41467_2020_17377_MOESM1_ESM.pdf]

Supplementary Information for: The emergence of magnetic ordering at complex oxide interfaces tuned by defects

Park *et al.*

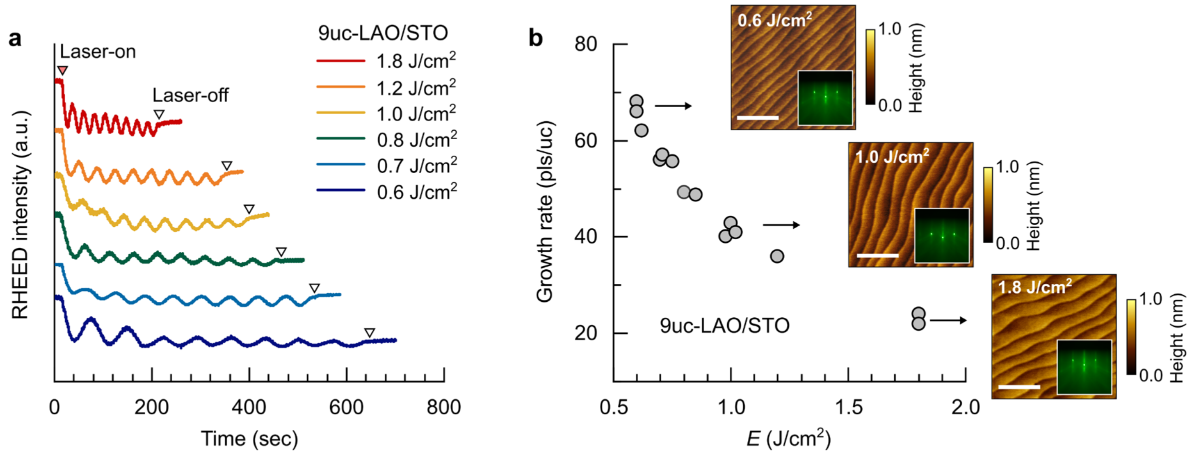

Supplementary Figure 1. a RHEED oscillations during the growth of the 9 uc-thick LAO thin films, grown on TiO_2-terminated STO(001), as a function of laser fluency (E), over the range of ~0.6 J/cm^2 – ~1.8 J/cm^2. **b** The laser fluency Vs growth rate of the LAO unit cell (uc). The inserts show the atomic force microscopy (AFM) images and RHEED patterns after the film growth and illustrate how atomically flat surface morphologies of all the grown 9uc-LAO/STO samples are. All scale bars represent 1 µm.

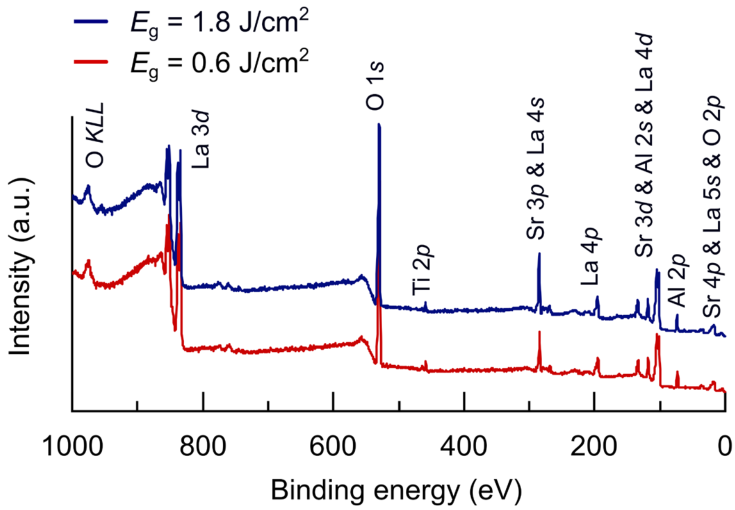

Supplementary Figure 2. XPS survey spectra of the LAO/STO samples grown by different laser fluencies, E_g = 0.6 J/cm^2 and 1.8 J/cm^2. No visible spectral weight and no difference for magnetic impurities were observed from the samples.

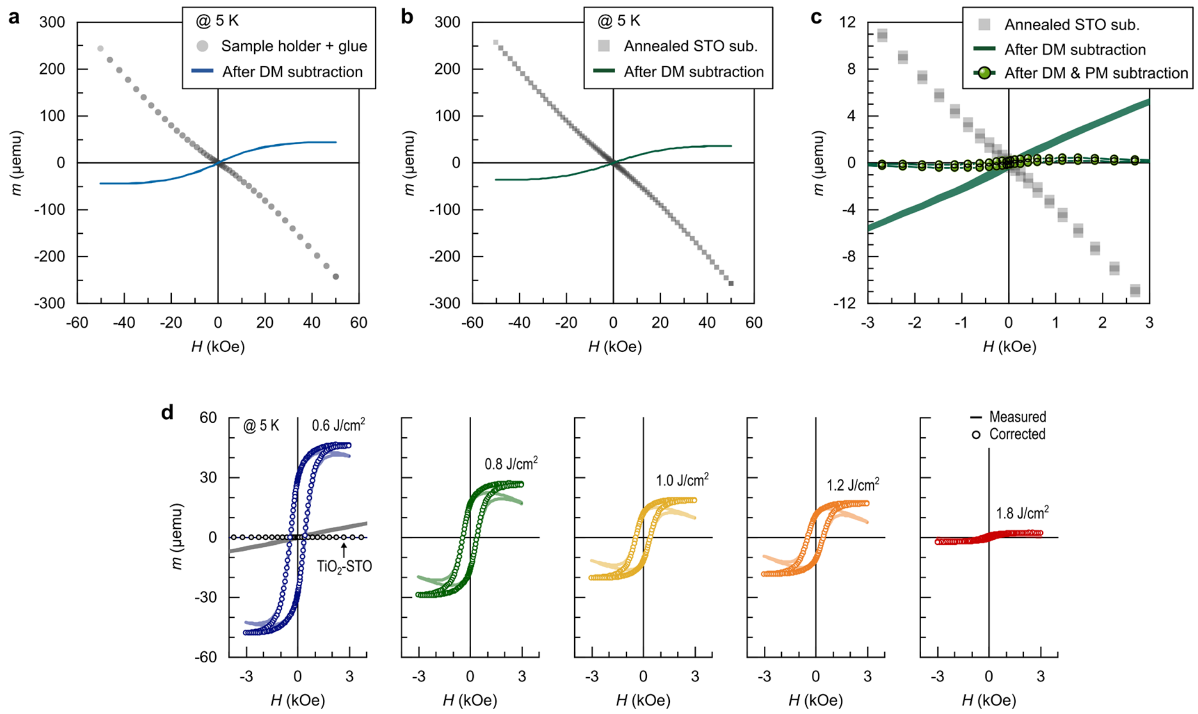

Supplementary Figure 3. a 5 K magnetic hysteresis loop of a sample holder, equipped in SQUID magnetometer, applying fields of +5 and -5 T. A paramagnetic (PM) background component was defined by subtracting the negative slope of a diamagnetic (DM) component. **b** 5 K magnetic hysteresis loop of an annealed TiO_2-terminated STO substrate mounted on the SQUID sample holder by low-temperature glue. **c** Hysteresis loop in the range of + and -0.3 T for the STO substrate. After subtracting the pre-existing DM and PM background components, a real magnetic hysteresis loop of the STO substrate indicates no ferromagnetism with measured moments of approximately 1 - 2 × 10^{-7} emu, which are not comparable with the measured moments of the LAO/STO samples. **d** 5 K magnetic hysteresis loops of the 9uc-LAO/STO samples as a function of E_g. The solid lines are the measured raw data, the dots are the corrected data after diamagnetic and paramagnetic subtraction.

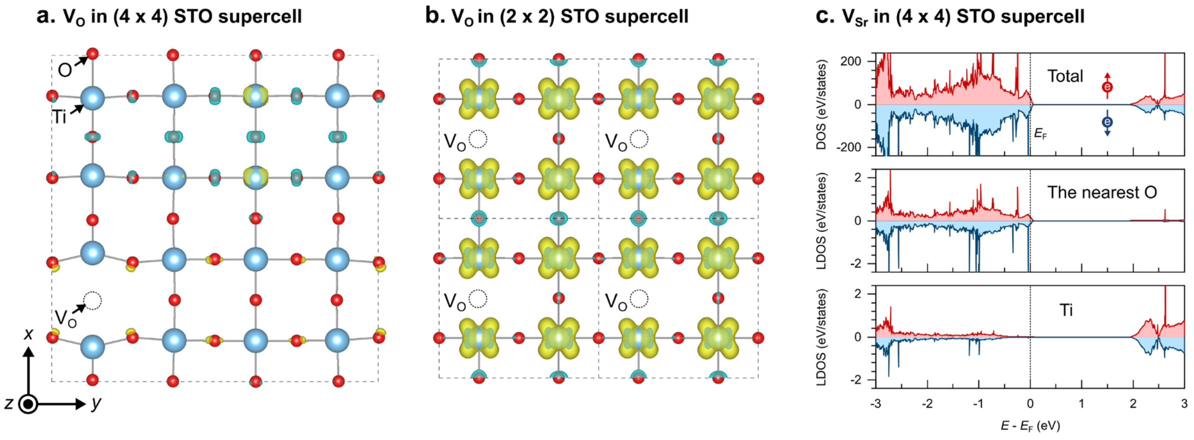

Supplementary Figure 4. a Magnetization density of a (4 × 4) STO supercell with an oxygen vacancy (~2 at.%), separated by 4*a* distance. *a* is the lattice constant of STO. The spin charge imbalance is given in yellow (positive) and cyan (negative) colour. **b** The magnetization density of a (2 × 2) STO supercell with oxygen vacancies (~8 at.%), separated by 2*a* distance. **c** Total spin-polarized density-of-states (DOS) for a (4 × 4) STO supercell with a Sr vacancy (in the upper panel) and the LDOS of the nearest neighbouring O (in the middle panel) and Ti (in the lower panel) atoms next to Sr vacancy.

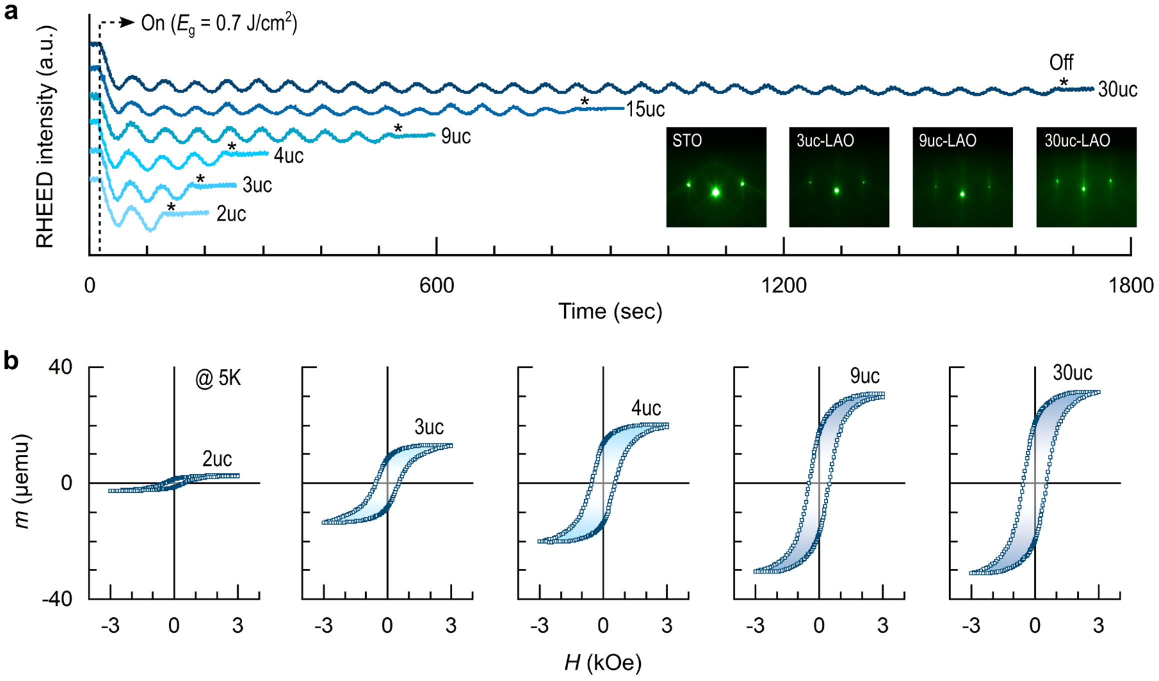

Supplementary Figure 5. a In-situ monitored RHEED intensity of B-site cation deficient LAO layers, grown on TiO_2-termainted STO(001) with thicknesses varying from 2 uc to 30 uc. **b** 5 K magnetic hysteresis loops of the B-site cation deficient LAO/STO samples with different LAO thicknesses.

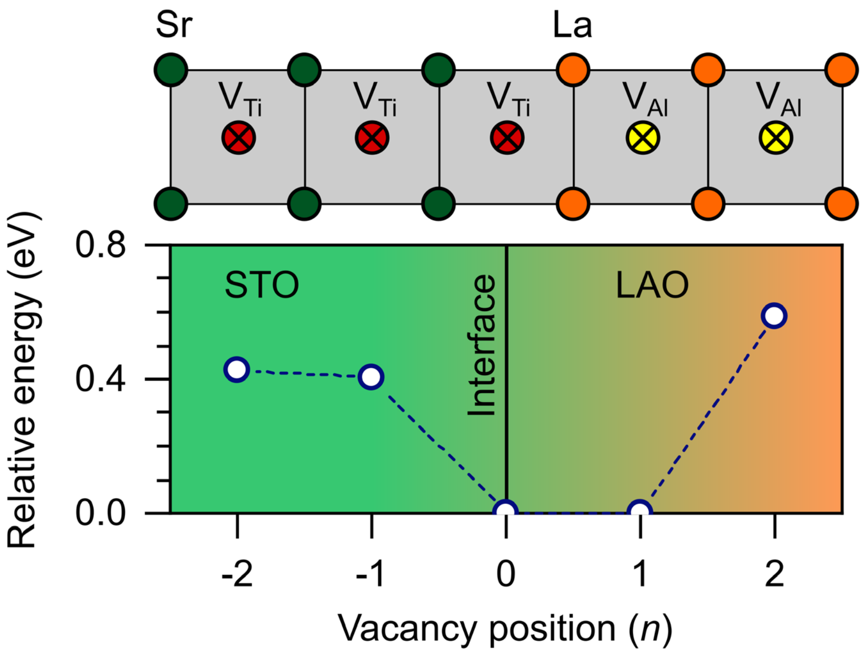

Supplementary Figure 6. The relative formation energy of V_{Ti} and V_{Al} near the LAO/STO interfaces with different layer positions, *n*. The formation energy of a B-site cation vacancy in each position was separately calculated. The relative energies were determined with respect to the energy of V_{Ti} (V_{Al}), positioned at the interface, $n = 0$ ($n = 1$).

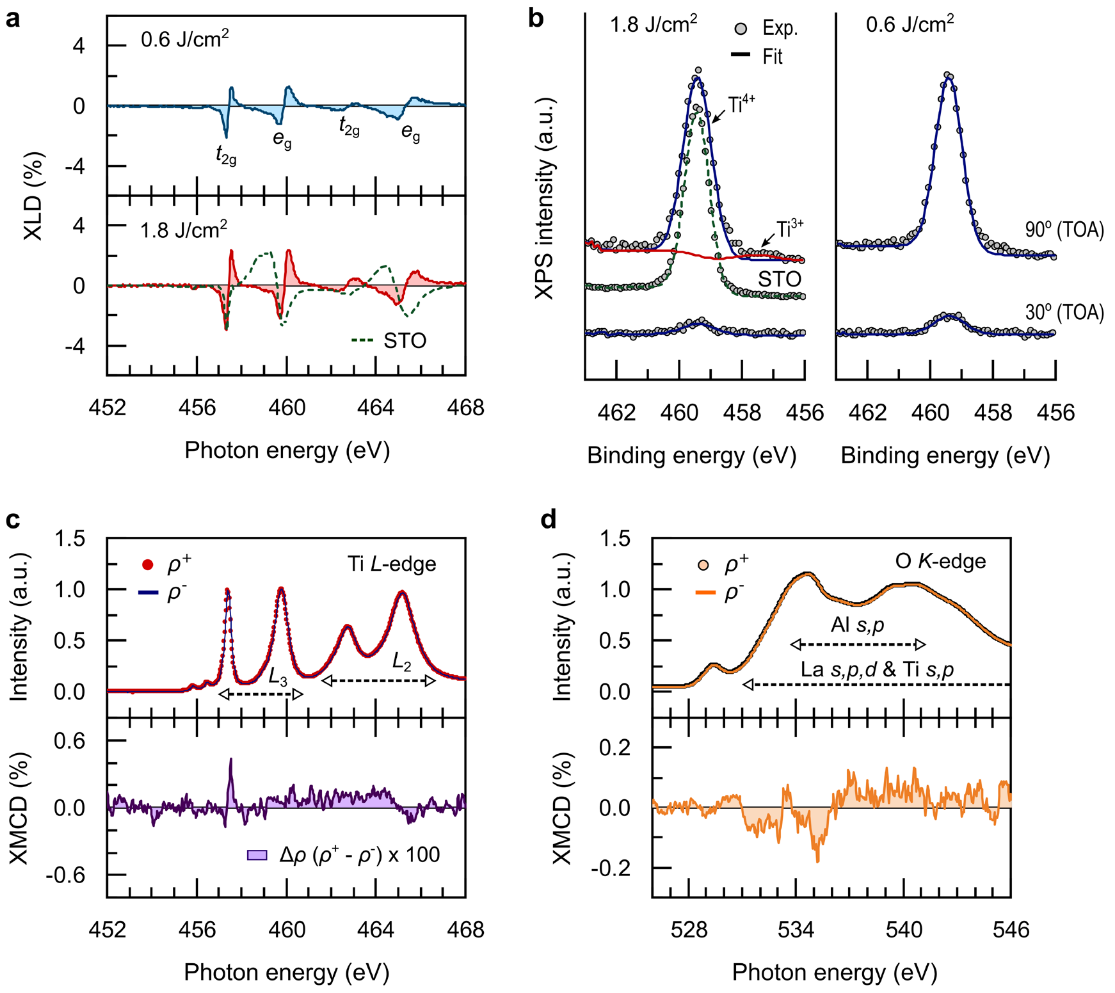

Supplementary Figure 7. a Ti *L*-edge X-ray linear dichroism (XLD) spectra of the B-site cation deficient (0.6 J/cm2) and B-site cation rich (1.8 J/cm2) LAO/STO samples. **b** XPS Ti 2*p* core-level spectra of the samples, taken with varying TOAs of 90 degrees (the maximum probing depth of approximately 6.0 nm) and 30 degrees (approximately 3.1 nm). **c** 2 K X-ray absorption spectra of the B-site cation deficient LAO/STO (0.6 J/cm2) for Ti *L*-edges, measured using circular polarized light with the photon spin parallel (ρ+) or antiparallel (ρ-) with respect to the magnetic field (in the upper panel), and the corresponding X-ray circular dichroism (XMCD) spectrum (in the lower panel). **d** RT X-ray absorption spectra of the B-site cation deficient LAO/STO for O *K*-edges, measured using circular polarized light (in the upper panel), and the corresponding XMCD spectrum (in the lower panel).

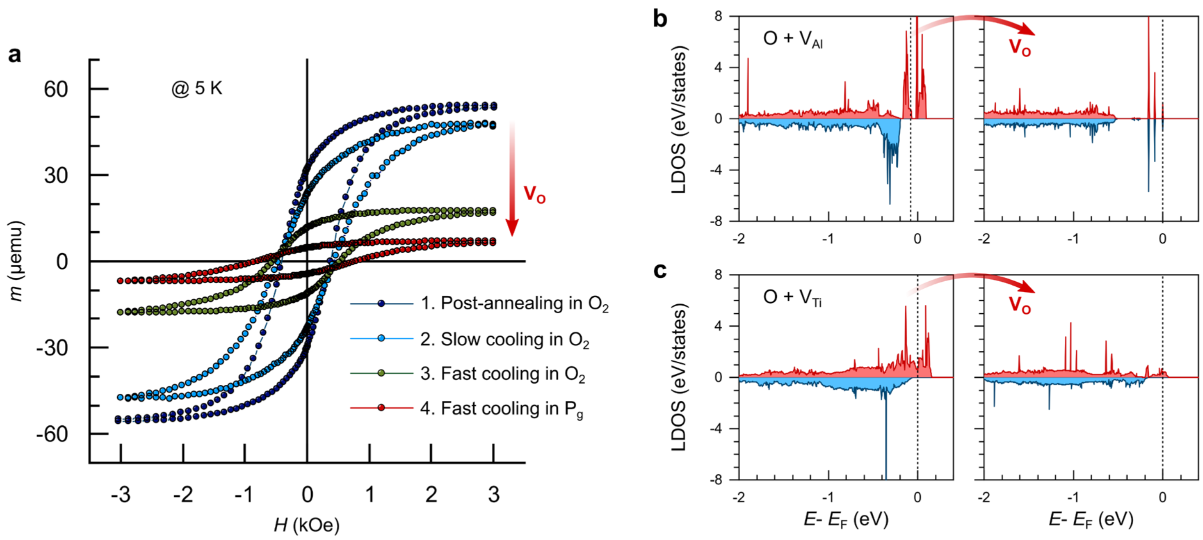

Supplementary Figure 8. a 5 K magnetic hysteresis loops along [100] of the B-site cation deficient LAO/STO samples grown by the laser fluency of 0.6 J/cm^2. To clarify the effect of oxygen vacancy on the interfacial magnetism, variations in the magnetic moments of the samples were controlled by altered cooling-down conditions after film growth: (1) post-annealing at 500 °C in O_2 (200 mbar) for 1 hour, (2) cooling-down in O_2 (200 mbar) with a rate of 10 °C/min, (3) cooling-down in O_2 (200 mbar) with a rate of 30 °C/min, and (4) cooling-down in growth oxygen partial pressure ($P_g = 5 \times 10^{-4}$ mbar) with a rate of 30 °C/min. **b** Calculated local density-of-states (LDOS) near the valence band states for the neighboring six oxygen atoms next to a V_{Al} in LAO (in the left panel) and the PDOS of the neighboring six oxygen atoms next to a V_{Al} with a V_O (in the right panel). **c** Partial density-of-states (PDOS) near the valence band states for the neighboring six oxygen atoms next to a V_{Ti} in STO (in the left panel) and the PDOS of the neighboring six oxygen atoms next to a V_{Ti} with a V_O (in the right panel).

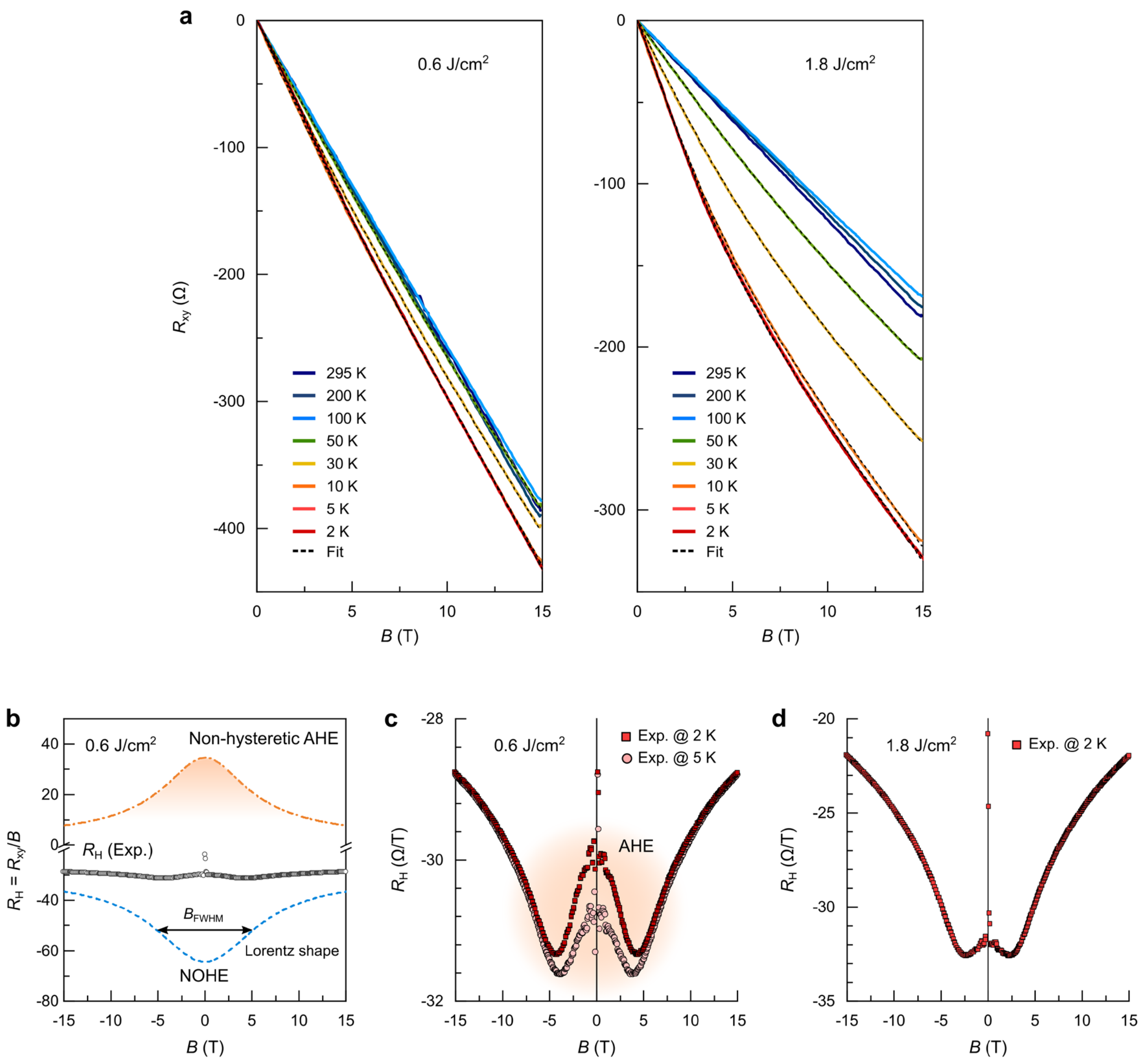

Supplementary Figure 9. a Variable temperature R_{xy} of the B-site cation deficient (0.6 J/cm^2) and rich (1.8 J/cm^2) 9uc-LAO/STO samples. The experimentally obtained data were fitted by using the extended model considering the contribution of both the NOHE and non-hy steretic AHE, denoted by the dashed lines. **b** NOHE and non-hysteretic AHE fitting parts of the experimental Hall coefficient, R_H (= R_{xy}/B), of a B-site cation deficient LAO/STO sample (E_g = 0.6 J/cm^2), measured at 2 K. The AHE of the sample can strongly perturb the NOHE over the applied field: the AHE part of R_H increases toward the zero field, opposite to the Lorentz-shaped NOHE part. **c,d** Experimental Hall coefficient, R_H [$=R_{xy}$ (Exp.)/B], of the B-site cation-deficient (**c**) and -rich (**d**) samples.

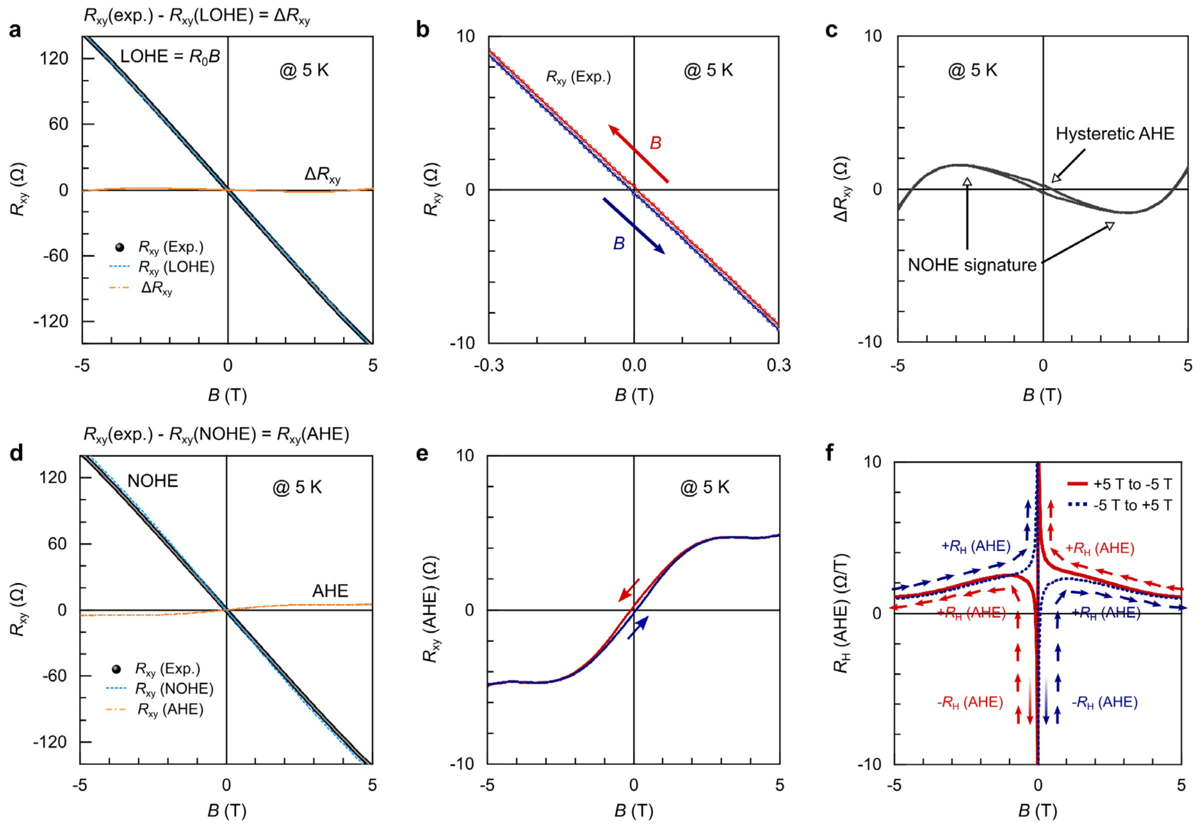

Supplementary Figure 10. a R_{xy}(LOHE) and residual R_{xy} (ΔR_{xy}) parts of the experimental 5 K-Hall resistance [R_{xy} (Exp.)] of a B-site cation deficient LAO/STO sample, grown with E_g = ~0.6 J/cm^2, measured in a magnetic field of 5 T with a field sweep of 1 mT/sec. ΔR_{xy} part is obtained after subtracting the LOHE part ($R_{xy} = R_0B$) to the total R_{xy} (Exp.). **b** The remanent R_{xy} (Exp.) of the sample at the zero field while sweeping the fields. **c** Residual *S*-shaped R_{xy} over the applied field and hysteretic R_{xy} in small field range. **d** NOHE and AHE fitting parts of the R_{xy} (Exp.) of the sample. **e** The deconvoluted hysteretic AHE part and the difference of the forward and reverse R_{xy} (Exp.) while sweeping the fields at 5 K. **f** The hysteretic AHE part of the R_H (Exp.) of the sample, measured in the field range of 5 T. The hysteretic AHE part visibly appears in the applied field below 1 T. Correspondingly, a spiky negative R_H occurs while the applied fields cross over the zero field and the negative R_H remains up to B ~40 - 50 mT.

Supplementary Note 1: Surface-sensitive X-ray photoelectron spectroscopy.

The total intensity, $I(E)$, of photoelectrons which are emitted from a distance, L, below the surface can be given by the Beer-Lambert law [1]:

$$I(E) = I_0(E)\exp(-\frac{L}{\lambda sin\theta_{TOA}}),$$

where $I_0(E)$ is the initial intensity of the electrons with energy, λ is the inelastic mean free path (IMFP) of the electrons, and θ_{TOA} is the photoemission angle (the polar angle of the detector to the surface: a take-of-angle).

The photoelectron IMFP in the materials can be approximated by using a modified IMFP formula, chiefly, the TPP-2M formula which considers the bandgap (E_g), atomic/molecular weight (M), density (ρ), and stoichiometry of the materials [1]:

$$\lambda(E) = -\frac{E}{E^2[\beta \ln(\gamma E) - (C/E) + (D/E^2)]}, (1,1)$$

where

$$\beta = -0.10 + 0.944/(E_p^2 + E_g^2)^{1/2} + 0.069\rho^{0.1}, (1,2)$$

$$\gamma = 0.191\rho^{-0.50}, (1,3)$$

$$C = 91.097.1U, (1,4)$$

$$D = 8.204.53U, (1,5)$$

$$U = N_v\rho/M = E_p^2/829.4, (1,6).$$

Here, $E_p = 28.8(N_v\rho/M)^{1/2}$ is the free-electron plasmon energy (in eV), ρ is the density (in g/cm^3), N_v is the number of valence electrons per atom (for an element) or molecule (for a compound), M is the atomic or molecular weight, and E_g is the bandgap energy (in eV). Equations (1,1) via (1,6) are known as the TPP-2M equation [1]. At normal emission, a photoemission signal of 65 % (95 %) is within λ (3λ) of the surface. A typical escape depth of a photoelectron, excited by Al $K\alpha$ source (hv = 1486.6 eV), is significantly less than 10 nm in an energy-dependent universal curve of λ. Since the effective path length to the surface can be obtained and predicted by the effective IMFP, $\lambda_{eff} = \lambda \cdot \sin\theta_{TOA}$, the depth-dependent chemical composition at the surface are obtainable by varying the incident photon energy and take-of-angle. This surface sensitive technique was applied to determine the atomic composition of the grown LAO films using a θ_{TOA} = 30 degree, corresponding to a probing depth of approximately

λ_{eff} = 1.05 nm (3λ_{eff} = 3.15 nm). Thereby, the determined compositional ratios of the elements (e.g. La, Al, Sr, and Ti) mostly reflect the chemical composition of the 9 uc-thick (~3.4 nm) film layer of the samples. In addition, the elemental composition is determined by the close-neighbour Al 2*p*, La 4*d*, Sr 3*p*, and Ti 2*p* core-levels as it is more accurate in terms of electron kinetic energy and escape depth (related to the IMFP of photo-excited electrons) [1,2].

Supplementary Note 2: DFT calculations on the magnetic properties of O and Sr vacancies in STO.

DFT shows that Vo-induced magnetism in STO strongly depends on the concentration of Vo for the magnetization of Ti d_{xy} orbitals as shown in Supplementary Figures 4a,b. In a (4 × 4) STO supercell, the magnetic moment of a single Vo (~2 at.%), separated by the distance of 4*a* (where *a* is the lattice constant) becomes a negligible value of 0.003 μ_B/Vo. In contrast, a higher Vo concentration (~8 at.%), formed as a regular sublattice in STO by separating the distance of 2*a*, effectively magnetizes Ti d_{xy} bands, similar to other reported results. This results in 0.4125 μ_B/ Vo after relaxation. Recently, a similar feature on the magnetic double exchange interactions between magnetic species Ti^{3+} and Ti^{4+} was demonstrated [3]. However, the Vo concentration in the modeled structure seems unrealistically high. Also, practically this is not our case as all the strongly magnetic B-site cation deficient LAO/STO samples were oxidized after film growth.

To investigate the magnetic effect of the Sr vacancy in STO, a (4 × 4) STO supercell model structure with a Sr vacancy was calculated by DFT. Unlike B-site cation vacancy effect, there is indeed a very weak A-site cation vacancy effect ($m < 0.07$ μ_B/V_{Sr}) on the creation of magnetism of STO (Supplementary Figures 4c) although the defective system electronically shows a *p*-type characteristic. This could be due to much less charge rearrangement of neighbouring oxygens and cations nearby Sr vacancy by the following factors: (*i*) only two electrons can contribute to neighbouring oxygens by Sr vacancy sites, compared with four in the case of Ti (4+) cation vacancy; (*ii*) the Sr site has 12 nearest oxygens at relatively larger distance of 2.76 Å, whereas Ti has 6 oxygen neighbours at significantly shorter distance of 1.95 Å. Therefore, Sr vacancy (or A-site cation vacancies in ABO_3 perovskites) would not dramatically change the electronic structure. This eventually results in the magnetic moment of each oxygen atom, less than 0.001 μ_B.

Supplementary Note 3: X-ray absorption spectroscopy measurements.

To shed light on the electronic and magnetic structures of the B-site cation deficient LAO/STO interface, X-ray absorption spectroscopy (XAS) measurements were performed at the Ti $L_{2,3}$-edges (transitions of Ti $2p_{3/2}$ to $3d$ states) and the O K-edge (O $1s$ to $2p$ states mixed with the energy states of all the constituent atoms) for both the B-site cation rich (1.8 J/cm^2) and deficient (0.6 J/cm^2) LAO/STO systems. Supplementary Figure 7a shows the Ti L-edge linear dichroism in the XAS spectra of the samples, obtained by subtracting the in-plane XAS intensity from the out-of-plane XAS intensity. These spectra directly correspond to the variation in the crystal field of the TiO_6 moieties at the interfaces, enabling the energy splitting between the in-plane and out-of-plane $3d$ electronic states of the Ti ions to be determined. The results show that both sides of the interface have a pronounced anisotropy of the Ti $3d$ levels, opposite to the dichroism of bare STO. Such a dichroism feature at the LAO/STO interface is typically owing to the symmetry breaking and orbital reconstruction of the Ti d bands at the polar/nonpolar interface, consistent with previous results in other reports, the energy of the planar orbitals (d_{xy} and $d_{x^2-y^2}$ in the Ti t_{2g} and e_g bands) is lower than that of out-of-plane orbitals ($d_{yz,xz}$ and d_{z^2}) [4]. This is attributed to a distortion of the TiO_6 octahedra, driven by either the dipole field of the polar LAO upper layer and/or charge transfer or V_O accumulation near the interface. In this work, we have confirmed that the orbital reconstruction near the interface is driven primarily by the built-up potential of the LAO upper layers and according to our findings both the insulating (Al-deficient) and conducting (Al-rich) LAO/STO interfaces, which are completely oxidized, show almost identical lifted-degeneracy of d-orbitals for the Ti^{4+} ions. However, the insulating B-site cation deficient interface shows a much lower (around 50 %) dichroism integral than that of the conventional LAO/STO interface, which corresponds to a lower spectral fraction of the Ti^{3+} state in the XPS (Supplementary Figure 7b). These arise from a reduced electrostatic potential at the B-site cation deficient LAO layer through the interfacial relaxation, as weaker electronic reconstruction and charge confinement occurs at the oxidized interface. It was predicted that insulating LAO/STO interfaces are formed exceeding 1/6 Al vacancies per uc ($\geq$ 33 %) in the interfacial AlO_2 layer. In this work, the lowest Al deficiency is determined to be approximately 16.2 % by XPS. The reduced dipole field of the B-site cation deficient LAO layer by the cation defect distribution still drives enough sites to form a tetragonal distortion and the subsequent orbital reconstruction of TiO_6 at the interface, although the electrical conducting state diminishes [5,6].

We performed X-ray magnetic circular dichroism spectroscopy (XMCD) at the Ti $L_{2,3}$ (measured at 2 K) and O K-edges (measured at RT) to clarify the magnetic structure of the oxidized B-site cation deficient LAO/STO interface. The spectra were collected in grazing incidence with the magnetic field (B = 6 T) along the in-plane

direction forming an angle of 20° with the film surface (the maximum probing depth of approximately 3 nm). Intriguingly, the Ti $L_{2,3}$-edge XMCD measurements exhibit prominent dichroism ($\Delta\rho$ of the total absorption signal) at a photon energy of 457.5 eV, as illustrated in Supplementary Figure 7c, while a broad and asymmetric dichroism feature in the O K-edge was observed at RT (Supplementary Figure 7d). The 457.5 eV sharp dichroism reflects the in-plane magnetization of the t_{2g} orbitals of the Ti^{4+} ions, with respect to the anisotropic linear dichroism (Supplementary Figure 7a). No spectral features related to Ti^{3+} ions can be observed and the spectral shape of our XMCD spectrum is very similar to the pure Ti^{4+} spectrum reported by Saluzzo *et al.* [7]. This observation also confirms that there is minor or no impact by Ti^{3+} on the magnetism of the oxidized B-site cation deficient interface.

Furthermore, provided that oxygen ions are themselves are spin-polarized when surrounding the cation defects, the broad small spectral feature of the O K-edge over a wide energy range reflects all of the Ti t_{2g} and e_g-derived states, and the additional hybridized O 2p states with unoccupied valence/conduction band states of the constituent metal ions. Such O-hybridization features were observed even at RT. It should be noted that the detection of Ti and Sr signals from the interface or the top surface of STO is partially hidden by the stronger signals from the O 2p states, hybridized with the La and Al empty bands from the LAO top layer. Thus, a reduced signal detection was predicted to be 1/9 of the signal from the 9uc-LAO layer. Nevertheless, surface-sensitive XMCD clearly indicates that the B-site cation deficient LAO/STO interface possesses exclusive magnetic components together with the Ti_{Al} antisite defect distribution in the Al-deficient LAO layer (Supplementary Figure 7b): the in-plane Ti d_{xy} orbitals and oxygen ions, are entirely dissimilar when compared with very weak Ti d_{xy} orbital–derived magnetism in conventional conductive LAO/STO interfaces [8].

Supplementary Note 4: Determination of nonlinear Hall resistance with two-carrier conduction and anomalous Hall effect.

Since the multiple carrier conduction in the LAO/STO interfaces results in the observed high-field nonlinearity of Hall resistance at low temperatures, T ≤ 50 K, two-electron populations were considered and the Hall resistance (R_{xy}^{2e}) is given by [9,10]:

$$R_{xy}^{2e} = -\frac{1}{e}\frac{[(n_1\mu_1^2/(1+\mu_1^2B^2))+(n_2\mu_2^2/(1+\mu_2^2B^2))]B}{[(n_1\mu_1/(1+\mu_1^2B^2))+(n_2\mu_2/(1+\mu_2^2B^2))]^2+[(n_1\mu_1^2/(1+\mu_1^2B^2))+(n_2\mu_2^2/(1+\mu_2^2B^2))]^2B^2} \quad (4,1)$$

with the constraint, $R_{xx}(B = 0) = e(n_1\mu_1 + n_2\mu_2)^{-1}$, where B, $n_{1,2}$, and $\mu_{1,2}$ are the applied magnetic field, the carrier density, and the carrier mobility of the two bands, respectively. Here, the non-linearity of the normal Hall effect from two-carrier case occurs, leading to a Lorentz-shaped R_H curve. The corresponding Hall coefficient can be expressed as:

$$R_H(B) = \frac{R_{xy}(B)}{B} = R_{xy}(\infty) + \frac{R_H(0)-R_H(\infty)}{1+(2B/B_{FWHM})^2}, \quad (4,2)$$

where $R_H(0)$ and $R_H(\infty)$ are the values of $R_H(B)$ at $B \rightarrow 0$ and $B \rightarrow \infty$ limits, respectively. The B_{FWHM} also describes the full width at half maximum of the Lorentz-shaped R_H curves.

$$B_{FWHM} = 2\frac{n_1\mu_1+n_2\mu_2}{(n_1+n_2)\mu_1\mu_2}. \quad (4,3)$$

For LAO/STO, the Hall resistance is generally *S*-shaped and the overall shape of the non-linear Hall resistance is governed by the presence of two *n*-type bands (d_{xy} and d_{xz}/d_{yz}) with different electronic properties. The experimental Hall resistance (R_{xy}) data are deconvoluted into two contributions from the two-band conduction and the anomalous Hall effect (AHE), as given by:

$$R_{xy} = R_{xy}^{2e} + R_{xy}^{AHE} = R_{xy}^{2e} + R_0^{AHE}M_z(B), \quad (4,4)$$

where M_z is a spontaneous magnetization in the *z*-direction in the LAO/STO interfaces. An additional correction term describes the anomalous Hall component for the existence of the defect-associated magnetic ordering at the B-site deficient LAO/STO interfaces. A field-dependent non-hysteretic AHE can be described by a Langevin-type expression as $R_{xy}^{AHE} = R_0^{AHE}\tanh(B/B_c)$, where R_0^{AHE} is the resistance with the saturation magnetization, M_0, and B_c is the critical magnetic field for the moment alignment [10]. Note that this functional expression is only valid to describe a non-hysteretic AHE, used for fittings of the symmetized non-hysteretic Hall resistance data (Supplementary Figure 9).

The low-temperature non-linear ordinary Hall effect of both the B-site cation rich (E_g = 1.8 J/cm^2) and deficient (0.6 J/cm^2) LAO/STO samples were fitted to the two-band model with the non-hysteretic AHE using the following conjectures (Supplementary Figure 9):

- For the B-site cation rich LAO/STO sample, the two-carrier conduction and non-hysteretic AHE model at 2 K, two-carrier conduction model at 5 K – 50 K, and linear ordinary Hall effect at 70 K – RT were employed.
- For the B-site cation deficient LAO/STO sample, the two-carrier conduction and non-hysteretic AHE model at 2 K – 50 K, and linear Hall effect at 70 K – RT.

To clarify the coupling effects between the defect-induced magnetic moments and the 2DEG near zero field, magnetotransport measurements of a B-site cation-deficient LAO/STO sample were carried out with refine field intervals (1 – 2 mT/sec) considering the measurement time (/field interval) dependence of the ferromagnetic coercive strength of the system. As seen in Supplementary Figure 10, a clear hysteretic R_{xy} appears at the zero field while sweeping the fields. This corresponds to an increase in the Hall coefficient, R_H, towards zero field and a spiky negative R_H then occurs when the applied field is reversely switched across the zero field due to the magnetic remanence of the system.

Supplementary References

1. Hofmann, S. Auger- and X-ray photoelectron spectroscopy in materials Science, *Springer Berlin Heidelberg*, 2013.
2. Tanuma, S., Powell, C. J. & Penn, D. R. Calculations of electron inelastic mean free paths. V. Data for 14 organic compounds over the 50–2000 eV range. *Surf. Interface Anal.* **21**, 165 (1994).
3. Doenning, D. & Pentcheva, R. Control of orbital reconstruction in $(LaAlO_3)_M/(SrTiO_3)_N(001)$ quantum wells by strain and confinement. *Sci. Rep.* **5**, 7909 (2015).
4. Salluzzo, M. *et al.* Orbital reconstruction and the two-dimensional electron gas at the $LaAlO_3/SrTiO_3$ interface. *Phys Rev. Lett.* **102**, 66804 (2009).
5. Warusawithana, M. P. *et al.* $LaAlO_3$ stoichiometry is key to electron liquid formation at $LaAlO_3/SrTiO_3$ interfaces. *Nat. Commun.* **4**, 2351 (2013).
6. Gunkel, F. *et al.* Charge-Transfer in B-site-depleted $NdGaO_3/SrTiO_3$ heterostructures. *APL Mater.* **6**, 076104 (2018).
7. Salluzzo, M. *et al.* Origin of interface magnetism in $BiMnO_3/SrTiO_3$ and $LaAlO_3/SrTiO_3$ heterointerfaces. *Phys. Rev. Lett.* **111**, 087204 (2013).
8. Lee, J.-S. *et al.* Titanium d_{xy} ferromagnetism at the $LaAlO_3/SrTiO_3$ interface. *Nat. Mater.* **12**, 703 (2013).
9. Gunkel, F. *et al.* Defect control of conventional and anomalous electron transport at complex oxide interfaces. *Phys. Rev. X.* **6**, 031035 (2016).
10. Christensen, D. V. *et al.* Strain-tunable magnetism at oxide domain walls. *Nat. Phys.* **15**, 269 (2019).
